# Exploring challenges in audiovisual translation: A comparative analysis of human- and AI-generated Arabic subtitles in *Birdman*

**DOI:** 10.1371/journal.pone.0311020

**Published:** 2024-10-21

**Authors:** Islam Al Sawi, Rania Allam

**Affiliations:** Faculty of languages, October University for Modern Sciences and Arts, Cairo, Egypt; Shanghai International Studies University - Songjiang Campus, CHINA

## Abstract

Movies often use allusions to add depth, create connections, and enrich the storytelling. However, translators may face challenges when subtitling movie allusions, as they must render both meaning and culture accurately despite existing language and cultural barriers. These challenges could be further complicated by the use of available AI tools attempting to subtitle movie allusions, while probably unaware of existing cultural complexities. This research investigates these challenges using qualitative and descriptive quantitative approaches by analyzing the movie *Birdman or (The Unexpected Virtue of Ignorance)*, comprising13.014 words, to identify the types of allusions used and compare the human- vs. AI (ChatGPT)-generated Arabic subtitles in terms of the subtitling strategies, their frequency, and quality. The results revealed that the movie used 52 Noun Phrase (NP) allusions, where the writer intertextually employed a proper name to convey meaning, and 8 Key-Phrase (KP) allusions, where the writer used phrases that convey implicit meaning easily perceived by members of the source culture (by referring to religious, literary, or entertainment texts). For NP allusions, both the human translator and AI opted for retentive strategies; however, the human translator’s preference to add guidance/parentheses to mark NP allusions was distinct. Additionally, it was observed that AI used neologism to render technology-related allusions, which could be a suggested strategy for NP subtitling into Arabic. For KP allusions, while the human translator seemed to be cognizant of the idea that KP allusions typically require a change in wording, AI fell short. Specifically, the human translator employed reduction in 5 out of 8 KPs, opting for minimum change/literal translation only three times. Conversely, AI utilized literal translation in all 8 examples, despite its awareness of the allusion and its intricate meaning/reference. As for the FAR assessment, for NP allusions, it revealed minor semantic errors in AI’s subtitles that did not affect the plot. Regarding KP allusions, AI’s subtitles were penalized in 5 out of its 8 Arabic renditions, in contrast to the human translator. Most of the errors were serious semantic errors that likely disrupted the flow of reading the subtitles due to conveying irrelevant meanings in the movie’s/scene’s context. Despite its functionality, this study suggests adding an extra parameter to the FAR model: consistency, as it plays a role in enhancing audience involvement and understanding. Its absence, as observed in some AI instances, can be misleading.

## Introduction

Today, Machine Translation (MT) has become a highly active research area, attracting significant interest across various fields [1]. This is true for Audiovisual Translation (AVT), where the integration of AI has opened new avenues for research [2]. AVT, whether in the form of subtitling or dubbing, has gained significant scholarly attention due to its unique combination of verbal, visual, and acoustic elements [3, 4]. This research explores the intersection of AI and AVT by comparing human and AI-generated subtitles, focusing specifically on the challenges associated with translating allusions from English to Arabic in audiovisual media.

Subtitling, one of the primary modes of AVT, involves converting spoken language into written text on-screen, enabling audiences to access content across language barriers [5]. It is particularly prevalent in the Arab world, where subtitled content is commonly broadcast on television and streaming platforms such as MBC2 and Netflix [6, 7]. The subtitling process is constrained by the need to synchronize with visual and acoustic elements while maintaining the original message’s intent and tone [8].

One of the significant challenges in AVT is the translation of allusions, which are references to people, places, customs, or events that carry cultural or contextual significance [9, 10]. These references can be particularly difficult to translate due to their reliance on shared cultural knowledge, which may not be familiar to the target audience [11]. In the context of Arabic subtitling, this challenge is further compounded by linguistic and cultural differences, requiring careful consideration of translation strategies to ensure accuracy and accessibility and acceptability [12–14].

Despite the advancements in AVT, there remains a noticeable gap between academic research and industry practices, especially in the translation of culturally specific content like allusions [15]. This study aims to address this gap by examining how allusions in the film *Birdman or (The Unexpected Virtue of Ignorance)* (hereafter referred to as *Birdman*) are rendered by both human translators and AI, specifically ChatGPT. ChatGPT is noted for its capabilities in general task processing, machine translation, and natural language understanding, making it a pertinent tool for examining AI’s role in AVT [16].

This research will analyze the types of allusions used in *Birdman* and compare the subtitling strategies employed by human translators and ChatGPT, evaluating their effectiveness based on [17–19]. These models provide a framework for identifying, categorizing and assessing functional equivalence, acceptability, and readability of translated content, which are critical metrics in evaluating subtitle quality. The study aims to answer the following research questions:

1. What allusions are present in *Birdman*?
2. What translation strategies did the human translator and ChatGPT use to render these allusions, according to [17, 18] typology?
3. To what extent are the subtitled allusions functionally equivalent, acceptable, and readable based on [19] FAR model?

## Review of related literature

This section reviews previous research related to allusions in AVT and ChatGPT to underscore the need for our paper and highlight existing gaps. We start by contextualizing ChatGPT’s relevance to AVT and focusing specifically on the translation of allusions.

ChatGPT, released by OpenAI in November 2022 [20], is based on large language models (LLMs), specifically the GPT-3 series, and later improved with the GPT-4 series. This architecture surpasses earlier models like recurrent neural networks (RNNs) and convolutional neural networks (CNNs) [21]. ChatGPT excels in various Natural Language Processing (NLP) tasks, including language understanding, text generation, and machine translation [22]. Its advanced capabilities in contextual understanding, language generation, and multilingual proficiency render it a promising tool for machine translation [22]. However, despite these advancements, the quality of ChatGPT’s translations, especially for low-resource languages like Arabic, remains questionable. This challenge arises from the limited availability of comprehensive parallel corpora and the intricacies of designing effective prompts [23]; even human “translators who operate in the English/Arabic language combination and translation scholars confront substantial hurdles when it comes to the availability of parallel corpora” [24: 2]. This sets the stage for our study, which investigates how ChatGPT handles the translation of allusions in Arabic subtitles compared to human translators.

### ChatGPT related translation studies

First, translation studies have unraveled the importance of integrating AI in translation research studies to study its potential and limits. [25] conducted an evaluative study on the aptitude of ChatGPT for Arabic-English MT, comparing its performance against other MT systems, such as Google Translate, intricately tailored for translation purposes. The study mainly sought to gauge ChatGPT’s translation efficacy in comparison with human translation expertise. The study compared the translation of a comparable corpus of 1000 English sentences from the open online platform Tatoeba and their equivalent Arabic ones translated by MT systems together with a human translation reference. Upon evaluating the translation quality produced by ChatGPT and Google Translate using the BLEU (Bilingual Evaluation Understudy) metric, a marginal advantage of ChatGPT over Google Translate was detected, which leads to the need to investigate ChatGPT’s ability to render allusions in AVT material.

Along the same line [26], conducted a study which aimed to examine the contrasts between human and AI translation on a collection of legal contracts, investigating the hypothesis that there was now no difference between both translations, and thus AI may lead to less need for human translators. The paper also attempted to assess full dependence on AI translation, namely ChatGPT. To achieve this, the legal texts were given to legal translators to translate and fed into the ChatGPT application, to compare both translations. The study concluded that, “AI translation can be faster and more cost-effective than human translation, however, it may not always capture the subtleties and cultural nuances of the text as accurately as a human translator can” (p. 11). Besides, despite the great leaps achieved by AI translation lately, providing timesaving, cost-effective options, it still needed many advancements. From the contextual, cultural, and lexical levels, the translation quality of a skilled human translator for legal texts could be more accurate than ChatGPT translation quality, especially in what concerns the legal terminology of different jurisdictions and concepts. The study thus recommended assigning a skilled, expert human translator in legal translation to ensure the highest levels of accuracy, especially with idioms and other linguistic features, confirming the need for further studies on using AI for various translation tasks. Additionally [27], investigated the accuracy of ChatGPT translations for media texts from Arabic into English to detect the strategies employed by professional translators in post-editing such translations. The results of the study pinpointed that the ChatGPT translations for Arabic-English media texts were not reliably accurate and needed post-editing by human translators, employing mainly three strategies: addition/expansion, omission/semantic minimization, and playing with diction.

These studies collectively emphasize the relevance of AI, particularly ChatGPT, in translation research and highlight the need for further studies on its effectiveness in subtitling allusions.

### Related studies on subtitling allusions

Several studies have focused on the subtitling of culturally specific items by human translators [12–14, 28]; however, studies on allusions and their translations from English to Arabic as distinct “cultural bumps” are scarce, despite representing translational complexities [29]. However, very few studies have investigated the subtitling of allusions from English to other languages.

[30] conducted a study analyzing the strategies employed to subtitle allusions from English into Turkish. He employed [18] strategies of translating allusions to quantitatively probe the allusive elements in The Simpsons Movie, aiming to determine whether the translation leans towards source text (ST) faithfulness (foreignization) or target text (TT) acceptability (domestication). The study concluded that the retention strategy was favored over replacement and omission in the translation of proper name allusions. For key phrase allusions, literal and standard translation strategies were the most used. He concluded that allusions were mainly kept unchanged, faithful to the source language (SL), adopting ‘foreignized’ subtitling strategies for allusions in the movie. This approach may leave target readers perplexed by several cases of ‘culture bumps,’ thus rendering the movie boring and tasteless. Therefore, there is a need for further studies to investigate more effective strategies that translators may use to render allusions through AVT.

[31] investigated the translation of allusions in movies employing Relevance Theory by comparing two Chinese versions of The Simpsons Movie. They concluded that allusion subtitle translation “is considered as a process of achieving Optimal Relevance (OR)” between the allusions in subtitles and the target readers (TRs). They identified several translation techniques used in subtitling allusions of The Simpsons Movie. For proper name (PN) allusions, five techniques were identified: retaining the name in its target language (TL) form, retaining the name as such while adding further explanation, retaining the name in its conventional TL form and adding extra information to clarify the allusion, replacing the name with a common name in TL form to achieve or without keeping the allusion, and omitting the name but translating the sense by its meaning. For key phrase (KP) allusions, six techniques were identified: resorting to literal translation when the allusion was familiar to the TR, replacing the KP with its standard TL form, adding additional information to describe the KP allusion, inserting further explanation to supplement the KP allusion when time and space limits allowed, reducing the allusion to its basic meaning by rephrasing, and omitting the allusion when songs were used to imply a certain milieu in some scenes that did not need translation. These findings confirm the need for culturally aware translators who can effectively handle the rendition of allusions between cultures, and the need for further research, especially when the TT is Arabic.

## Methodology

This study adopts a descriptive-analytical approach to explore the subtitling strategies used for allusions in the movie *Birdman* and their translation into Arabic. By examining the original English allusions and their corresponding Arabic subtitles, the study aims to analyze how different types of allusions are translated and the strategies employed. The analysis is conducted using established frameworks discussed in “The framework”.

### The movie and subtitles

*Birdman* is an American satirical movie that was released in 2014 and won four Academy Awards. It centers around Riggan Thomason (played by Michael Keaton), a washed-up actor who was once famous for a superhero movie titled “Birdman”. The movie attempts to draw the audience behind the scenes and into Thomason’s mind, making it quite complex and quirky. However, the movie was hailed as a masterpiece by many critics internationally [32], making it worth studying. Renowned for its complexity and critical acclaim, the movie was selected for its intricate narrative and rich use of allusions. The Arabic subtitles for *Birdman* were extracted from the Disney+ streaming service, known for its provision of professionally subtitled content, ensuring high-quality material for analysis.

### The framework

Allusion can be defined as “a reference which is indirect in the sense that it calls for associations that go beyond mere substitution of a referent” [33: 521]. It is employed “to instruct an audience, to generate an aesthetic experience in an audience, and to link or connect the author with a tradition by activating themes, motifs, and symbols” [33: 521]. Thus, it is there to be detected by the intended source recipient (SR) due to common cultural exactitude, yet it needs extra exposition of unfamiliar target recipient (TR). To analyze allusions in *Birdman* and their subtitling into Arabic, three frameworks were employed: [17] model for NP allusions [18], framework for KP allusions, and [19] FAR model.

### Ren’s (2024) NP model

**Fig 1:**
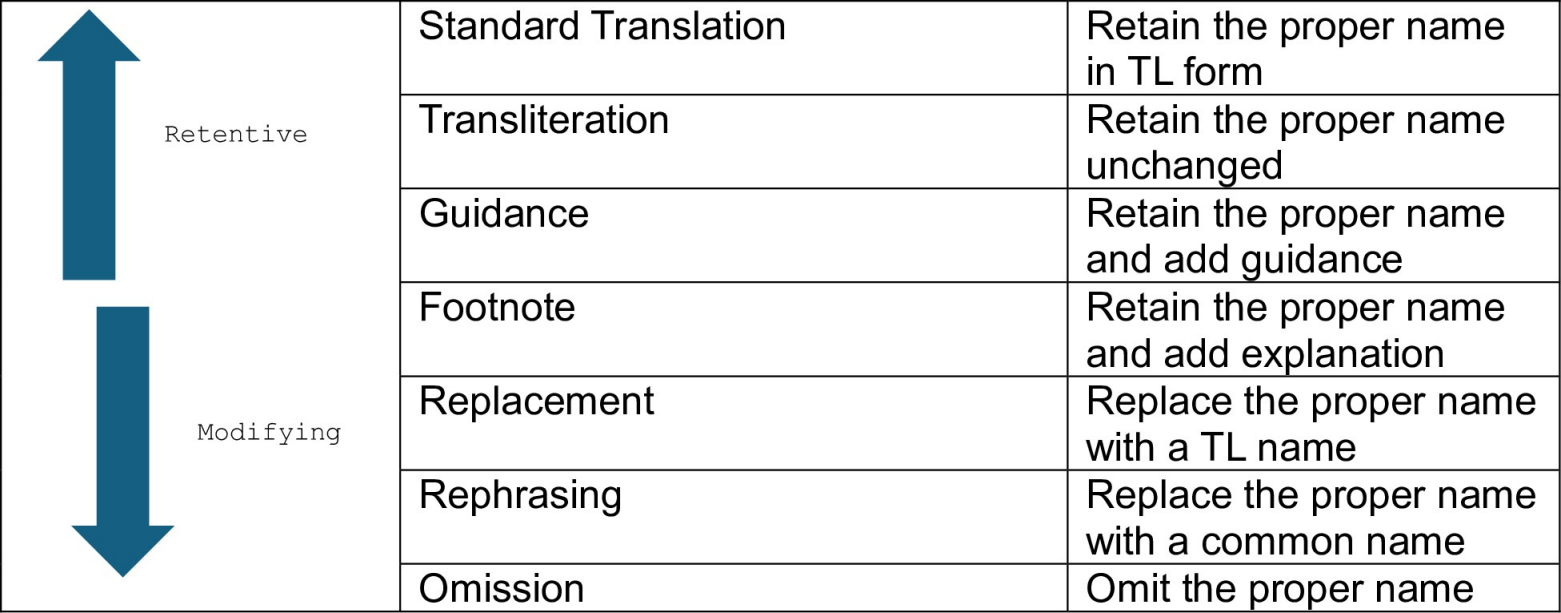
Illustration for Ren’s modified model for NP allusion translation strategies.

Ren’s (2024) framework, a modern adaptation of [18, 34] models incorporating transliteration strategies, was chosen for its relevance in translating proper name allusions. This model builds on earlier frameworks by integrating recent strategies for handling orthographical differences between English and Arabic, thereby enhancing the recency and applicability of the research (Fig 1).

### Leppihalme’s (1997) framework for KP allusions and minimax ordering for rendering KPs

**Fig 2:**
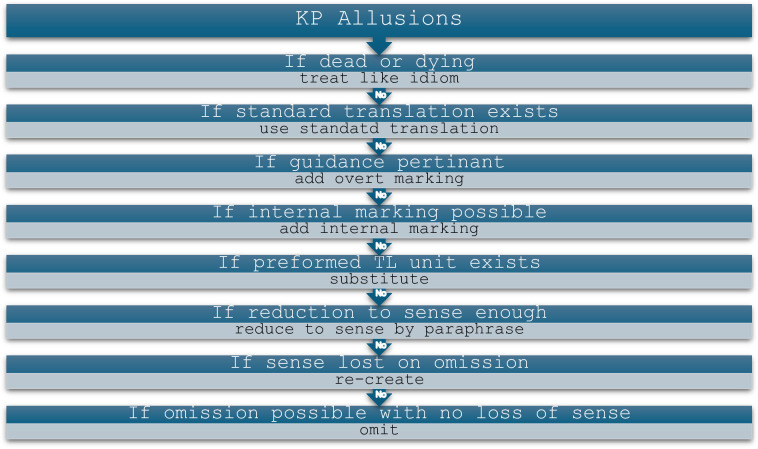
An illustration for Leppihalme’s (1997) minimax ordering for rendering KPs.

Leppihalme’s (1997) framework helps identify and analyze KP allusions and suggests a minimax ordering for subtitling KP allusions which can help better understand the decisions made by translators whether human or AI. KP allusions refer to phrases that convey implicit meaning easily perceived by members of the source culture (by referring to religious, literary, or entertainment texts), but which convey little or no meaning when translated verbatim to the target audience. These phrases pose significant challenges for translators because they presuppose a level of audience participation based on familiarity with the phrase and its connotations [18]. Consequently, translators must consider how to render these phrases for the target audience and make conscious decisions accordingly. Leppihalme added that translators would follow a minimax ordering for rendering KP subtitling as shown in (Fig 2). Our research investigates whether this minimax ordering is observed by translators, whether human or AI, when rendering movie allusions from English to Arabic (Fig 2)

### The FAR model

The acronym FAR stands for Functional Equivalence, which refers to the effective rendering of meaning from the ST to the subtitled TT; Acceptability, which relates to the degree to which the subtitles meet the TT norms; and Readability, which refers to how easy the TT subtitles are for the audience to understand. The FAR model proposed by [19] is found suitable to assess the quality of the subtitles under study because, as the name suggests, “it looks at renderings of languages that are not “near” to you (i.e., your own) but “far” from you (i.e., foreign)” [19: 217]. Additionally, it has been used in several studies to assess subtitle quality related to culturally specific terms [12, 28].

The FAR model is based on error analysis and awards penalty points depending on the nature of the error and its effect. Thus, errors are identified as minor, where the error may go unnoticed; standard, where the error may ruin the subtitle for the audience; and serious, where an error may affect the understanding of the subtitles and subsequent ones because of it being so blatant that the audience may take longer to resume automated reading of the subtitles. It is worth mentioning here that if the translator only renders the meaning of ST and avoids verbatim renderings, it is then not an error but rather a preferred subtitling practice. It is an error when only what is said is rendered devoid of meaning because that can be considered misleading.

Equivalence errors are classified into two types: Semantic and Stylistic. Semantic errors’ penalty points are as follows: minor: 0.5, standard: 1, and serious: 2. A Semantic error is minor when it is lexical and hence does not affect the plot of the movie. A Semantic error is classified as standard when the error still has relevance to the TT meaning and does not affect subsequent subtitles. A Semantic error is serious when it disturbs the flow of reading subtitles and affects following ones. On the other hand, Stylistic errors, such as using wrong terms of address, register, or style, are not as serious as semantic errors because they do not usually cause misunderstandings.

Acceptability relates to the degree to which the subtitles meet the TT norms. So, errors in this area occur when the subtitle sounds foreign or unnatural and are classified into 1) grammar errors, 2) spelling errors, and 3) errors of idiomaticity. Grammar errors can be minor or standard if they do not affect meaning, but a serious grammar error is one that may make the subtitle difficult to read and understand. Spelling errors are classified as minor when they do not change the meaning of the TT word; standard, when there is a change of meaning; and serious, when the word is impossible to read. It is relevant to our study here to further highlight idiomaticity errors in this model. It does not only refer to using idioms but rather to the natural use of an idiom that is native to the ST and film audience. Errors in this area are not grammatical but relate to source text interference. If the subtitle’s idiomatic use is erroneous but does not affect understanding and is just unnatural, it can be categorized under acceptability, but if it is too serious that it hampers understanding, it should be under equivalence issues.

**Fig 3:**
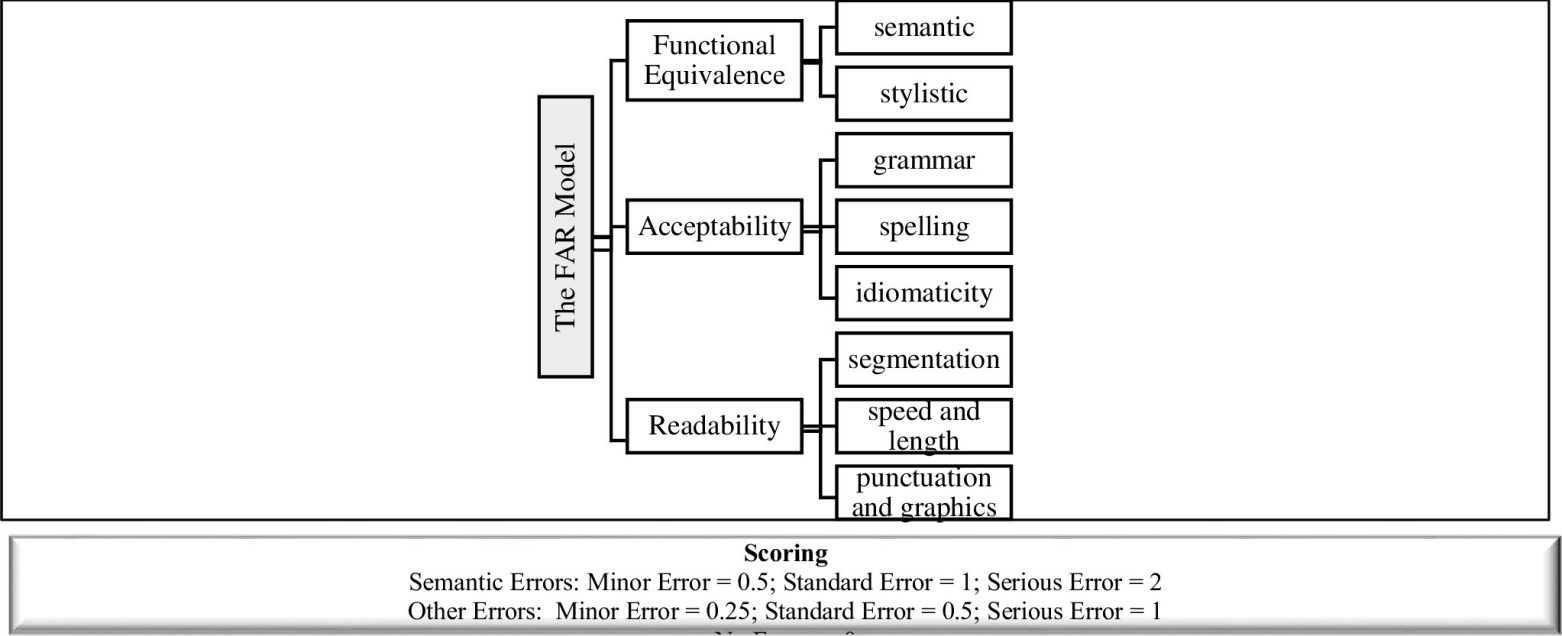
The FAR model.

Readability relates to technical errors and to reading the text effortlessly. They are categorized into errors of segmentation and spotting, punctuation, reading speed, and line length. If the segmentation is unusual, synchronization with speech is lacking, or punctuation and graphics. In this category, serious errors are ones that relate to spotting when the subtitle is delayed from speech by more than one utterance. The FAR model was selected for its comprehensive approach to evaluating subtitle quality based on Functional Equivalence, Acceptability, and Readability. This model is appropriate for assessing errors in subtitling culturally specific terms, allusions in our case, and has been effectively applied in previous studies [12]. (Fig 3) summarizes the FAR model.

### Procedure

First, the movie *Birdman* and its Arabic subtitles were downloaded from the Disney+ platform. Then, allusions were identified and categorized into NP and KP allusions based on their contextual and cultural relevance. Afterward, the ST allusions were given to ChatGPT (the free version, now ChatGPT-4 with limited access, which ensured using the latest and most accurate version of AI) in context to subtitle into Arabic. Following that, subtitling strategies were identified for both human and AI software by the two researchers once agreed. Finally, a FAR analysis was conducted to assess the quality of KP allusions. Because the two researchers are native speakers of Arabic, they were able to identify the errors and severity of subtitles. All Arabic in-text instances were accompanied by transliteration using the Library of Congress (LOC) system via (https://romanize-arabic.camel-lab.com).

## Results and discussion

### NP allusions

NP allusions, where the writer or author intertextually employs a proper name, constitute the most frequently used type of allusions both in general and in the data under analysis. In the dataset, 52 NP allusions referring to celebrities in the media industry, philosophers, and myths have been identified (See S1 Table).

**Fig 4:**
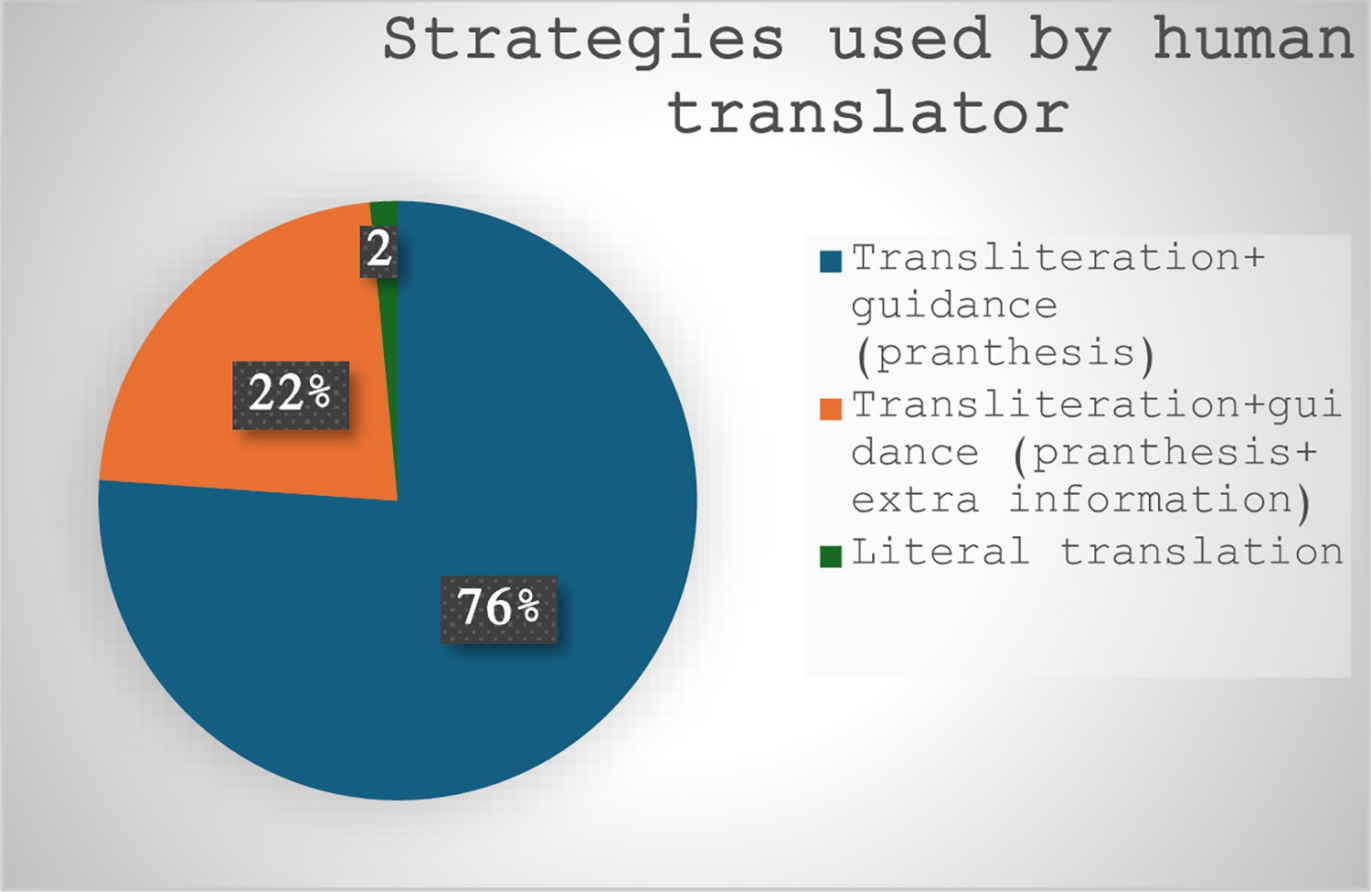
Strategies used by human translator.

**Fig 5:**
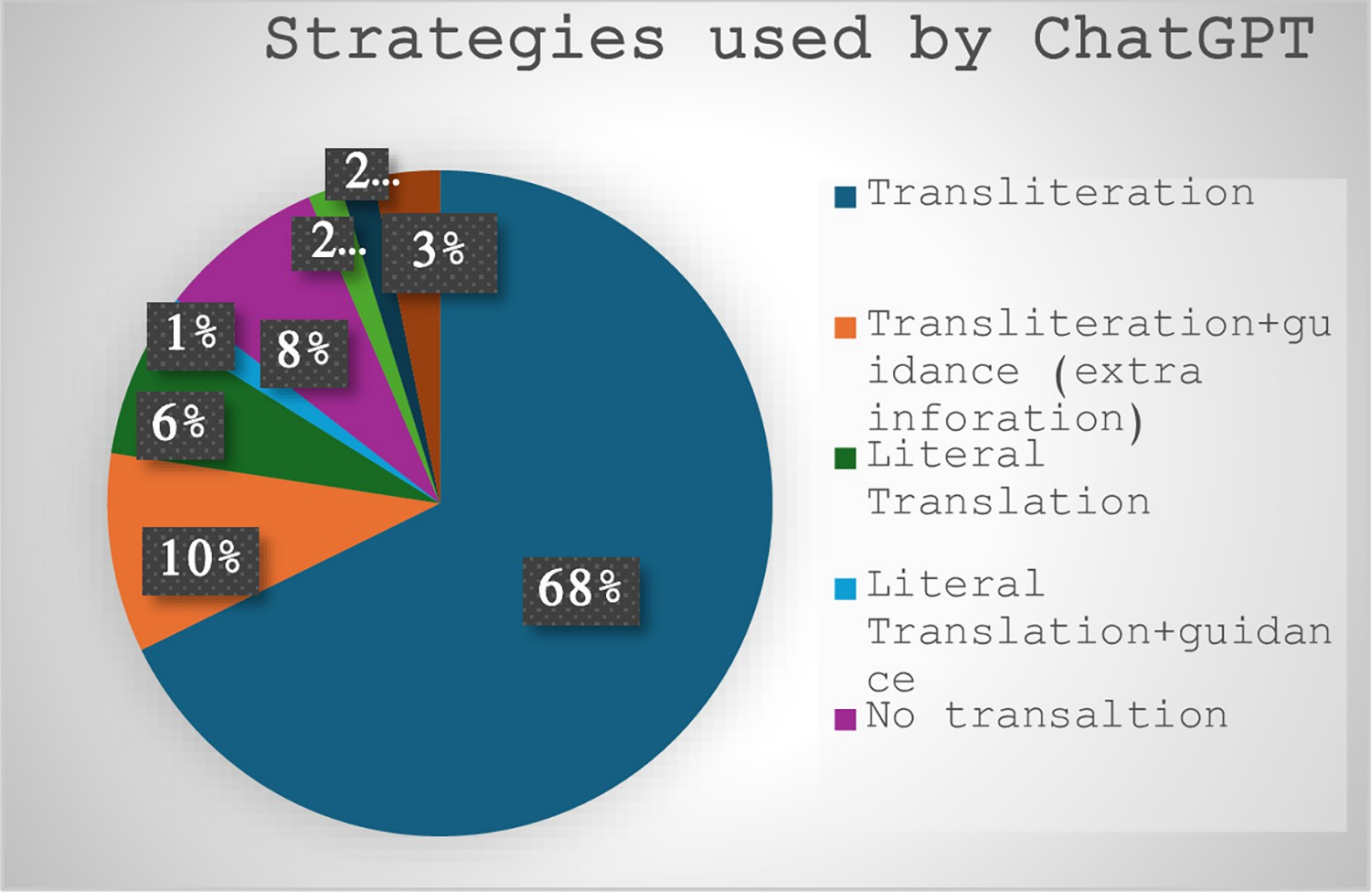
Strategies used by ChatGPT.

Using [17] most recent classification of possible strategies for rendering NP allusions, the human translator opted for transliteration 51 times and for literal translation 1 time, with all transliterations accompanied by guidance with parentheses marking the allusion in-text, and 16 by adding extra information to explicitly state what the allusion refers to. In comparison, ChatGPT utilized a broader range or strategies; it employed transliteration 41 times (6 supported by guidance, extra information), literal translation 4 times (1 supported by guidance, extra information), no translation 5 times (2 supported by guidance and inverted commas and 2 by inverted commas only), standard translation 2 times, neologism 1 time, and omission 1 time (by changing the proper name into common noun). It is worth mentioning here that literal translation is not considered a strategy according to Ren’s model. Previous studies indicated that literal translation of NP allusions is not a preferred strategy [17, 34]; however, it can be argued that, in our dataset, it was used successfully by the human translator to connote the ridicule played by the protagonist in the movie against superhero movies. Another strategy solely adopted by ChatGPT is using no translation, which refers to the machine retaining the English name(s) and marking them with inverted commas or inverted commas plus guidance (extra information). Both named strategies, along with other strategies and examples, are discussed after (Figs 4 and 5), which indicate numerically the percentage of adopted strategies by the human translator vs. AI.

(Fig 4) shows that the human translator chose transliteration and strategic marking of NP allusions with parentheses, as in “Raymond Carver to Birdman, like Icarus,” into “(بيردمان)، شأنه شأن (إيكاروس),” /(byrdmān), shaʼnuhu shaʼn (Īkārūs)/, and “He’s an Avenger,” into “إنه ممثل في (أفنجرز)” / Innahu mumathil fī (afnjrz)/. Additionally, he added some information to explain the allusions on 16 occasions where he used the word “فيلم” / Fīlm/ (film) 3 times, “مسرحية” /Masraḥīyah/ (play) 2 times, “تويتر” / Tūwītar /(Twitter) 1 time, “جريدة” /Jarīdah/ (newspaper) 1 time, “استعراض” /Istiʻrāḍ/ (show) 1 time, “اقتباس” /Aqtbās/ (quotation) 1 time, “شبيه” /Shabīh/ (double) 1 time, and “طبيب التجميل الخاص” /Ṭabīb al-Tajmīl al-khāṣṣ/ (her cosmetic surgeon) 1 time, “ممثل” /Mumathil/ “actor” 1 time, “رواية” /Riwāyah/ “novel” 1 time, “حساب” /Ḥisāb/ “account” 1 time, “مسرح” /Masraḥ/ “stage” 1 time, “صفحات” /Ṣafaḥāt/ “pages” 1 time, and “بطاقات” /Biṭāqāt/ “cards”1 time. The tendency to use retention translation strategies with NP allusions matches previous studies [17, 35]; however, a major difference here is consistently marking/guiding the audience that a “foreign” NP allusion is being used by using parentheses; it can be argued that this strategic marking helps lessen the “foreignness” of the target text and explicitly state the subtitles contain transliterated words. Finally, one strategy that was used by the human translator that was not a part of the adopted framework was literal translation where the translator chose to literally render “and he’s making a fortune in that Tin Man getup” into “ومع ذلك فهو يجني ثروة من ذلك الزي المعدني” /Wa-Maʻa dhālika fa-huwa yjny Tharwat min dhālika al-Zayy al-maʻdinī/ (And yet he makes a fortune from this iron getup). This choice could be explained by examining the probable use of this allusion in the ST. In the movie, the hero is referring to *Ironman* (an Avenger in Hollywood superhero movies) with the intention of criticizing superhero movies and ridiculing them. So, he uses the phrase “ذلك الزي المعدني” /Dhālika al-Zayy al-maʻdinī/ (this iron getup) to refer to *Ironman*. Hence, the human translator seems to stick to the same words to communicate the implicit meaning to the target audience. This preference was discussed by [36] where he said that translators may think about intended meaning and their audience when rendering a text into a target language.

As for ChatGPT, (Fig 5) reveals its broader ability to adopt several strategies to render NP allusions into Arabic. While the human translator opted for marking transliterated NP allusions, ChatGPT avoided this marking or the use of parentheses with NP allusions. Another major difference between the human translator and ChatGPT is in literal translation. To illustrate, while the human translator seems to be aware of the problems associated with the literal rendition of some NP allusions, which could result in mistakes or misunderstandings, ChatGPT appears to be doing that without a clear or valid reason. See (Table 1); these examples will be further elaborated on “Quality assessment of the allusions translated by human translator vs ChatGPT” when running a FAR assessment of the target translations.

**Table 1:** Example of faulty ChatGPT literal translations.

Time	English Text	ChatGPT subtitle	Strategy
**00:00:50.680**	(Late Fragment)	(الجزء الأخير)	Literal translation
**00:06:35.720**	He’s doing the next Hunger Games	هو يعمل على الجزء القادم من ألعاب الجوع	Literal translation
**00:48:04.160**	that dude from American Pie was there	تلك الشخصية من فيلم الفطيرة الأمريكي كان هناك	Literal translation+ guidance (extra information)
**01:04:49.720**	R. Kelly bad	آر كيلي سيء	Simple retention/transliteration+ literal translation

Another strategy that was used solely by ChatGPT is neologism, which refers to “a new word or expression, or a new meaning for an existing word” (Cambridge Dictionary, 2024). Table 2 shows how ChatGPT used the neologism “التغريد” /Altghryd/ (Tweeting) to render the source text allusion.

**Table 2:** ChatGPT use of neologism to render an allusion.

Time	English Text	ChatGPT Translation	Strategy
**00:09:37.720**	It was tweeted by @prostatewhispers	تم التغريد بواسطة @prostatewhispers.	Neologism +no translation

Based on that use, it may be suggested that this strategy be added to the strategies used for rendering allusions into Arabic, where a translator may have to render an ST NP allusion using neologism in the TL, especially when the term is intertextually referring to an invention, technology, or service. Another strategy that was solely used by ChatGPT was omission and replacement for “the Times” in “What do you think my friend, Tabitha is going to do to you in the Times?” to “ما الذي تعتقد يا صديقي، تابيثا ستفعله لك في الصحيفة؟” / Mā alladhī tʻtqd yā Ṣiddīqī, tābythā stfʻlh laka fī al-Ṣaḥīfah? / (What do you think my friend, Tabitha is going to do for you in the newspaper?). Omission is generally seldom used by translators when rendering allusions [17], which is also consistent with the results of analyzing the present dataset.

The analysis shows that, like previous studies [17, 34, 35], both the human translator and ChatGPT opt for retentive strategies probably to “avoid translation mistakes, ensure fluency, and improve the readability of the target text” [17: 14]. However, the human translator’s preference to add guidance/parentheses to mark NP allusions is different. Additionally, ChatGPT’s use of neologism could be another suggested strategy that enhances translators’ creativity and cultural sensitivity. This may also refer to translation strategies as suggestions that require further investigation and modification.

### Quality assessment of the allusions translated by human translator vs ChatGPT

Despite the FAR model only relating to assessing the quality of interlingual subtitling and may not directly apply to the subtitling of NP allusions that are normally transliterated into the ST, incidents of translation by other strategies whether by the human translator or ChatGPT can still be assessed using the model. The human translator exclusively used transliteration (whether with guidance with parenthesis or extra explaining information) except for one NP where s/he translated “and he’s making a fortune in that Tin Man getup” into “ومع ذلك فهو يجني ثروة من ذلك الزي المعدني” /Wa-Maʻa dhālika fa-huwa yjny Tharwat min dhālika al-Zayy al-maʻdinī/ (And yet he makes a fortune from this iron getup) where s/he uses literal translation successfully to communicate the denotative and connotative meaning of the ST allusion, and hence no errors were identified. In contrast, ChatGPT used a range of strategies: literal translation 4 times (1 supported by guidance, extra information), no translation 5 times (2 supported by guidance and inverted commas and 2 by inverted commas only), standard translation 2 times, neologism 1 time, and omission 1 time (by changing the proper name into common name). The FAR assessment revealed minor semantic errors that do not affect the plot of the film only with literal and no translation strategies; the viewers may still be able to know that it is a title of a movie or a name of a literary work or film (Table 3).

**Table 3:** Quality assessment of ChatGPT rendition of allusions.

Time	English Allusion	Arabic Subtitle (Machine)	Strategy	Functional Equivalence	Acceptability	Readability
**00:00:50.680**	**(Late Fragment)**	**الجزء الأخير)**)	Literal translation	Minor-Semantic- 0.5	0	0
**00:06:35.72**	He’s doing the next **Hunger Games**	هو يعمل على الجزء القادم من **ألعاب الجوع**	Literal translation	Minor-Semantic- 0.5	0	0
**00:06:46.00**	He’s **the Hurt Locker guy**. He’s **an Avenger**	هو الرجل الذي عمل على **فيلم "The Hurt Locker"** هو أحد أبطال **فيلم "The Avengers"**	Guidance (extra information) +no translation Guidance (extra information) +no translation	Minor-Semantic- 0.5 Minor-Semantic- 0.5	0	0
**00:09:37.72**	by **@prostatewhispers**	بواسطة **@prostatewhispers**.	no translation	Minor-Semantic- 0.5	0	0
**00:16:40.04**	I saw you in **Hothouse**	رأيتك في **"Hothouse"**	No translation	Minor-Semantic- 0.5	0	0
**00:30:09.36**	We should have done that reality show they offered us **The Thomsons**	كان يجب أن نفعل ذلك البرنامج الواقعي الذي عرضوه علينا **"The Thomsons".**	No translation	Minor-Semantic- 0.5	0	0
**00:48:04.16**	that dude from **American Pie** was there	تلك الشخصية من **فيلم الفطيرة الأمريكي** كان هناك.	Literal translation+ guidance (extra information)	Minor-Semantic- 0.5	0	0
**01:04:49.72**	**R. Kelly bad**	**آر كيلي سيء**	retention/transliteration+ literal translation	Minor-Semantic- 0.5	0	0

Another point we would like to add here and probably suggest for inclusion in the FAR model is consistency, which may be lacking in its current form. We observed instances where ChatGPT lacked consistency in rendering the same NP allusions in different events in the movie, seemingly without any obvious reason (Table 4). The movie/series *Avengers* was rendered with no translation as “the avengers” and with transliteration as “الأفنجرز” /Alʼfnjrz/ (the Avengers); The name “Barthes” was transliterated as “بارث” /Bārth/ and “بارت” /Bārt/. This lack of consistency was also reported by research on ChatGPT in various fields [37, 38]. However, in our dataset, it may not be considered very serious because it does not affect subsequent subtitles, reading speed nor understanding.

**Table 4:** ChatGPT’s instances of lack of consistency.

NP Allusion	ChatGPT rendition	NP Allusion	ChatGPT various rendition
He’s **an Avenger**	**“The Avengers”**هو أحد أبطال **فيلم**	with the equally successful **Avengers** series	سلسلة **أفلام الأفنجرز** التي حققت نجاحًا مماثلاً
Who is this **Barthes** guy?	من هذا الرجل **بارث**؟	**Roland Barthes** was a French philosopher	**رولان بارت** كان فيلسوفًا فرنسيًا

### KP allusions

We have identified 8 KP allusions in *Birdman*: 4 referencing literary works (1 to a poem and 3 to Shakespeare’s *Macbeth*), 2 idioms (considered by [18] as a linguacultural manifestation distinctive from allusion, but we consider them in audiovisual material as allusive texts that may cause cultural bumps if literally translated), 1 biblical reference, and 1 cultural allusion. (Table 5) presents the identified KP allusions along with their typology and suggested meanings/ references.

**Table 5:** KP allusions in *Birdman*.

Time/ Allusion	Type/Reference to	Meaning/reference
00:00:50.680 1. And did you get what you want from this life, even so? I did. And what did you want? To call myself beloved, to feel myself beloved on the earth.	Literary Allusion (Poetry)	The lines are from the poem *Late Fragment* written by the renowned American Writer Raymond Carver. The short poem presents the fundamental desire of humans for love and connection. That is, to love and be loved give meaning to life.
00:07:52.400 2. We handed these poseurs the keys to the kingdom	The Bible	In the Gospel of Matthew, Chapter 16, Verse 19, Jesus says to Peter, “I will give you the keys of the kingdom of heaven.” Combining this with the word “poseurs” suggests that insincere people were given significant power, often to the detriment of others.
00:16:59.440 3. Thank the Lord and pass the biscuits	idiom	The idiom reflects a blend of religiosity, gratitude, and hospitality commonly associated with Southern traditions.
00:23:41.760 4. He left a piece of his liver on the table every time he wrote a fucking page	idiom	A less common idiom used in the movie to refer to heavy drinking.
00:30:39.440 5. He said he has to be a redneck	culture	A stereotype of rural or Southern American culture, usually negative.
01:21:47.880 6. Tomorrow and tomorrow and tomorrow	Literary Allusion (Play)	It is the opening line of one of the most famous soliloquies in William Shakespeare’s play *Macbeth*. Macbeth, the protagonist, reflects on the passage of time and the meaningless nature of life, revealing a deep sense of hopelessness.
01:22:51.400 7. walking shadow	Literary Allusion (Play)	It is a line from William Shakespeare’s play *Macbeth*, where Macbeth, the protagonist, emphasizes the brevity and fragility of human existence.
01:23:10.200 8. It is a tale told by an idiot	Literary Allusion (Play)	It is a famous lime from William Shakespeare’s play *Macbeth*, where Macbeth, the main character, communicates a feeling of hopelessness and despair.

Having identified KP allusions and their references and meanings, we can confidently state that they convey deep meanings relevant to the movie’s storyline and the characters’ feelings. To elaborate, in *Birdman*, Riggan Thomson is a washed-up actor attempting to revive his career but struggling with his own battles with ego and experiencing strange occurrences that lead to a surreal conclusion. The KP allusions identified in (Table 5) illustrate the analogy between the troubled Riggan and Shakespeare’s Macbeth, while highlighting Riggan’s insecurities and desire for love and fame. Consequently, these KP allusions would necessitate conscious decisions from the translator to render them successfully to the target audience and maintain the allusion, or its connotation of deep loss and despair.

An analysis of the strategies used by human translation versus ChatGPT reveals that, unlike NP allusions, significant deficiencies in using various strategies to render allusions into Arabic in ChatGPT’s approach were identified. (Table 6) comparing the strategies used by human translation vs. ChatGPT demonstrates that human translation relied on reduction, minimal change, and literal translation, whereas ChatGPT predominantly employed literal translation, with one example of minimum change in the use of “الحمد لله” / al-Ḥamad lillāh / to render “Thank the Lord and pass the biscuits.”

**Table 6:** KP allusions translation strategy used by human translators Vs ChatGpt.

Allusion	Human Subtitle	Strategy	ChatGPT Subtitle	Strategy
1. And did you get what you want from this life, even so? I did. And what did you want? To call myself beloved, to feel myself beloved on the earth.	وهل حصلت على ما أردته من هذه الحياة، ولو من بعيد؟ أجل وماذا كنت تريد؟ أن أقول إنني محبوب، وأن أشعر بأنني محبوب على هذه الأرض	Minimum change	وهل حصلت على ما تريد من هذه الحياة، حتى الآن؟ نعم، حصلت. وماذا كنت تريد؟ أن أُعتبر محبوبًا، أن أشعر بأنني محبوب على هذه الأرض.	Literal translation
2. We handed these poseurs the keys to the kingdom	أعطينا لهؤلاء المتباهين مفاتيح النجاح	Reduction	سلمنا مفاتيح المملكة لهؤلاء المحتالة	Literal translation
3. Thank the Lord and pass the biscuits	حمدا لله!	Reduction	الحمد لله وتفضل بالبسكويت	Minimum change+ Literal translation
4. He left a piece of his liver on the table every time he wrote a fucking page	كان يشرب حتى الثمالة كلما كان يؤلف صفحة!	Reduction	ترك قطعة من كبده على الطاولة في كل مرة كتب فيها صفحة لعنتها	Literal translation
5. He said he has to be a redneck	قال إن عليه أن يكون قروياً	Reduction	قال إنه يجب أن يكون ريدنيك	Literal translation
6. Tomorrow and tomorrow and tomorrow	غداَ وغداَ وغداَ	Literal translation	غداَ وغداَ وغداَ	Literal translation
7. walking shadow	ليست الحياة إلا وهما	Reduction	الظل المتجول	Literal translation
8. It is a tale told by an idiot	الحياة قصة . . . يحكيها شخص غبي	Literal translation	إنها قصة ترويها أحمق	Literal translation

**Fig 6:**
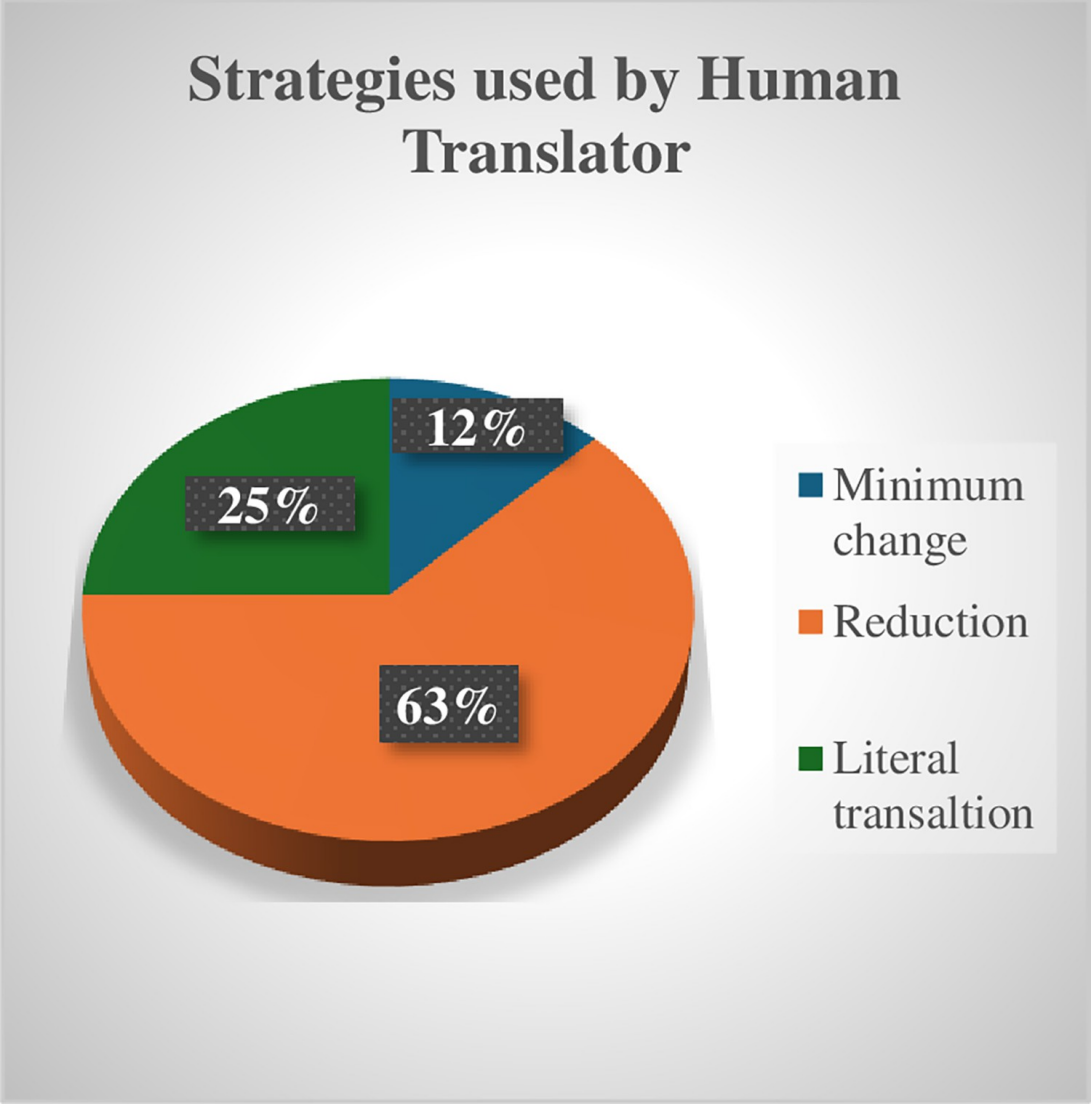
Strategies by human translator.

**Fig 7:**
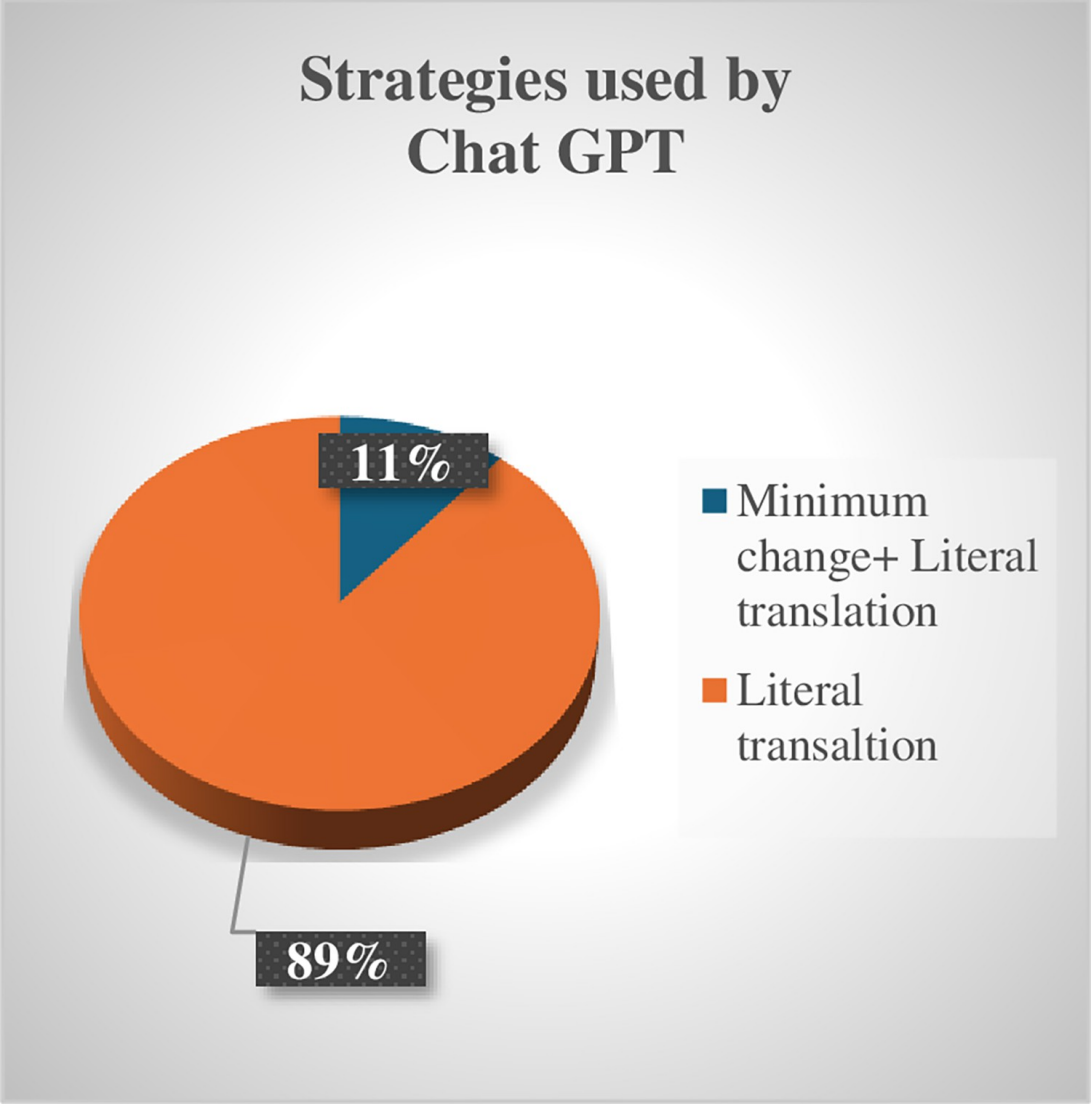
Strategies by ChatGPT.

**Fig 8:**
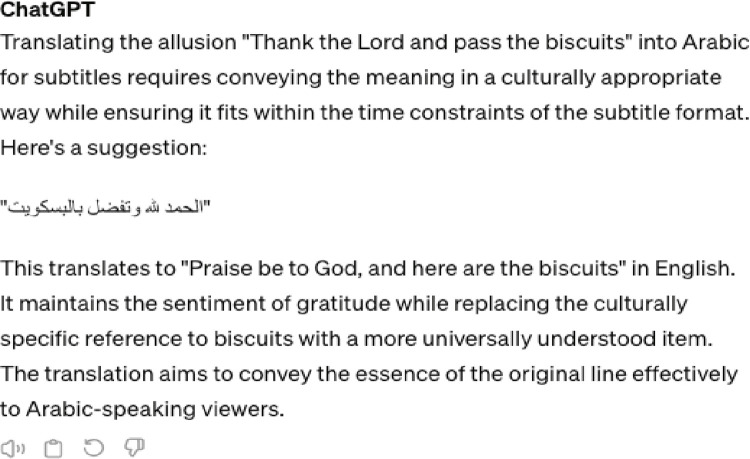
Excerpt chat between the writer and ChatGPT.

Unlike PN allusions, KP allusions typically require a change in wording [18]. While the human translator appears to be cognizant of this fact, ChatGPT falls short. Specifically, the human translator employed reduction in 5 out of 8 KPs, opting for minimum change/literal translation only thrice. Conversely, ChatGPT utilized literal translation in 8 examples, despite its awareness of the allusion and its meaning (see Fig 8 for the conversation between the researcher and ChatGPT). (Figs 6 and 7) summarize quantitively the strategies used by the human Translator vs ChatGPT to render KP allusions.

These results indicate that despite ChatGPT’s awareness, at least at a recall level, of KP allusions and available communicative translation strategies associated with them, it is unable to effectively convey them communicatively into Arabic. This inability is also known as hallucinations or insufficient modeling of explicit knowledge that result from errors and noise in the training data or other reasons beyond people’s and developers’ comprehension [16]. The question we need to explore now is why we perceive the strategies used by human translators as more appropriate than ChatGPT’s literal or minimal change strategies in dealing with the analyzed key points (KP allusions). This question can be analyzed in terms of [18] suggested minimax ordering of strategies for KP allusions (Fig 8).

### The human translator vs ChatGPT and Leppihalme’s minimax

Analysis of KP allusions demonstrates that while the human translator relies heavily on reduction to convey KP allusions into Arabic, ChatGPT predominantly resorts to literal translation, with only one instance of minimum change. According to [18], a proposed minimax ordering for rendering KPs should adhere to the steps outlined in (Fig 2).

(Fig 2) can help explain why the human translator predominantly opts for reduction. Some of the identified KP allusions, when translated literally, would result in awkward meaning in Arabic. For instance, the allusions “redneck” and “left a piece of liver on the table,” translated literally by ChatGPT as “ريدنيك” / Rydnyk / and “Tarak qiṭʻah min kbdh ʻalá al-ṭāwilah,” respectively, are meaningless in Arabic. It appears that while the AI tool failed to recognize this, the human translator was aware of the issue and thus considered alternative minimax ordering strategies for rendering allusions. They chose reduction by using “قرويً” / Qrwyun / and “كان يشرب حتى الثمالة,” / Kāna yashrab ḥattá althmālh,/ which convey the intended denotative meaning of the allusions. However, it is worth noting that despite successfully conveying the meaning/sense of the allusion, reduction may fail to communicate the connotative meaning implied by the allusion or alter it [39, 40]. For example, the word “قرويً,” / Qrwyun / used to render “redneck,” may connote different meanings for Arabic readers. In Egyptian Arabic, it may mean someone naive, or speaking a different accent/dialect but in this context, it refers to people with different characteristics, which could be offensive as in “an offensive term for a white person who is considered to be poor and uneducated, especially one living in the countryside in the southern US, who holds prejudiced beliefs” (dictionary.cambridge.org, 2024), which may not be captured in the chosen denotative reduction “قرويً,” / Qrwyun/. Similarly, despite the human translator effectively conveying the intended meanings echoed in Macbeth’s lines, the connotative meanings represented by recalling Macbeth’s internal conflict and dilemma diminishes among the Arabic audience. Hence, while ChatGPT fails to effectively communicate the meaning of the allusions into Arabic, the human translator appears to be adhering to the suggested minimax ordering for rendering KPs, opting to convey denotative meaning while sacrificing some connotations.

On another note, we observed that other available strategies were avoided by the human, which aligns with previous studies on the translation of KP allusions. These studies explained that translators often opt for retention, internal and external marking, rather than employing other strategies [30, 41]. Upon further examination of this preference [18], attributed it to translators not considering their target audience, feeling the need to exclude certain audience, being loyal to the source text, or having the urge to perform their job without interfering with the original work’s writing. Therefore, it is now valuable to assess the subtitles qualitatively using the FAR model.

### Quality assessment of the allusions translated by human translator vs AI

The FAR assessment of the human and ChatGPT subtitles reveals that 7/8 of the human translators” proposed subtitles did not score any penalty points; with some of them be rewarded if FAR model was also rewarding successful renditions (a major flow with frameworks based in penalizing errors) namely, 1 and 2 in (Table 7) where the human translator was able to locate the allusions, understand their connotative as well as denotive meanings, and render them in the ST. There was only a minor semantic error in an allusion from *Macbeth* where the human translator only rendered the denotative meaning, while ignoring the connotative one (Table 7, example 5). It is minor though because a part of the meaning was still existent, and it did not hamper understanding of the plot. In contrary to the human translator, ChatGPT’s subtitles were penalized in 5 of its 8 renditions to the KP allusions. Most of the errors were serious semantic errors that literally translated the KP allusion losing leading to probable disruption of the flow of reading the subtitles. There was only a minor spelling error, but it would not affect the message or the flow of reading. (Table 7) summarizes these mistakes.

**Table 7:** KP FAR assessment.

Allusion	Human Subtitle	Functional Equivalence	Acceptability	Readability	ChatGPT Subtitle	Functional Equivalence	Acceptability	Readability
1. Thank the Lord and pass the biscuits	حمدا لله!				الحمد لله وتفضل بالبسكويت	Serious Semantic: -2	0	0
2. He left a piece of his liver on the table every time he wrote a fucking page	كان يشرب حتى الثمالة كلما كان يؤلف صفحة!				ترك قطعة من كبده على الطاولة في كل مرة كتب فيها صفحة لعنتها	Serious Semantic: -2	0	0
3. He said he has to be a redneck	قال إن عليه أن يكون قروياً				قال إنه يجب أن يكون ريدنيك	Standard Semantic: -1	0	0
4. walking shadow	ليست الحياة إلا وهما				الظل المتجول	Serious Semantic: -2	0	0
5. It is a tale told by an idiot	الحياة قصة . . . يحكيها شخص غبي	Minor Semantic: -0.5			إنها قصة ترويها أحمق		Minor grammar: -0.5	0

ChatGPT’s inability to render many of the KP allusions into Arabic without errors is evident in (Table 7), a finding that seems contradictory to research papers suggesting using ChatGPT as a powerful translation tool [16].

## Conclusion

Allusions serve as references to extralinguistic, cultural elements, enhancing the depth and richness of AVT. However, they pose translational challenges when subtitled, as accurately conveying both meaning and cultural context is essential. These challenges become even more perplexing when addressed by AI Machine Translation (MT) tools. This research employed a mixed approach to analyze the movie *Birdman*. The mixed-method approach highlighted the value of employing diverse analytical frameworks to thoroughly understand translation strategies and challenges when subtitling movie allusions. Firstly, we identified and quantified the types of allusions in the movie, totaling 52 instances of NP allusions and 8 instances of KP allusions. The models proposed by [18, 34], and their modified version by [17] prove effective in discerning the translation strategies for rendering allusions in Arabic subtitles, whether adopted by human translators or AI. The analysis revealed an additional strategy employed by human translators for NP allusions, namely literal translation, which can be incorporated into Ren’s model as a practical strategy commonly used by translators. Furthermore, the study identified a strategy exclusively used by ChatGPT, namely neologism, which proved useful for tackling some allusions.

Overall, the analysis indicated a tendency for both human translators and ChatGPT towards retentive strategies for NP allusions in subtitling. However, the human translator’s preference for adding guidance or parentheses to mark NP allusions suggested a keen awareness of the need for further explanation to ensure acceptability by Target Audiences (TAs). In translating KP allusions, the human translator demonstrated linguistic skill and cultural sensitivity by adjusting the wording to convey the intended meaning from the source text (ST) effectively. In contrast, ChatGPT often failed to convey even the basic meaning accurately, resorting to literal translation in all examples despite being aware of the allusion and its meaning.

Regarding the FAR assessment, while primarily serving as an error analysis model for subtitles, it proved functional in evaluating the translation strategies of both translators. Minor semantic errors were observed in the translation of NP allusions by the human translator, while ChatGPT’s subtitles were penalized in nearly 62% of KP instances, classified as serious semantic errors that may distort the viewing experience for TAs. However, despite its functionality, this study suggested adding an extra parameter to the FAR model: consistency, as it played a crucial role in enhancing the TA’s involvement, linkage, and enjoyment.

In conclusion, this study underscores the need for further advancements in AI Language Model (LLM) translating devices, exemplified by ChatGPT, to achieve satisfactory translation quality, especially in contexts involving multisemiotic, linguacultural challenges. Future research could extend these assessments to larger AV corpora and diverse AV genres, while also exploring the capabilities of other AI MT tools to produce translations of varying quality levels.

## Supporting information

S1 Table NP allusions in English and Arabic with identified human and machine used strategies. (PDF)
