# Supplementary material for: Exploring challenges in audiovisual translation: A comparative analysis of human- and AI-generated Arabic subtitles in Birdman
Source: PLoS One. 2024 Oct 21;19(10):e0311020. doi: 10.1371/journal.pone.0311020 (PMC11493253; doi:10.1371/journal.pone.0311020)
Supplement: S1 Table — (PDF) [file pone.0311020.s001.pdf]

S1 Table. NP allusions in English and Arabic with identified human and machine used strategies.

Time	English Allsion	Type	Arabic Stubtitle (Human)	Strategy	Arabic Subtitle (Machine)	Strategy
**00:00:50.68**	**(Raymond carver, Late Fragment)**	proper name+proper name	(رايموند كارفر)، (لايت فراغمنت)	Simple retention/transliteration Simple retention/transliteration	(ريموند كارفر، الجزء الأخير)	Simple retention/transliteration Literal transatlion
**00:06:32.32**	Get me... **Woody Harrelson**	Proper name	(وودي هاريلسون)	Simple retention/transliteration + guidance (paranthesis)	احضر لي... ودي هارلسون!	Simple retention/transliteration
**00:06:35.72**	He's doing the next **Hunger Games**	Proper name	إنه يمثل في فيلم (هانغر غيمز) التالي	Simple Retention/transliteration+ guidance (extra information) + guidance (paranthesis)	هو يعمل على الجزء القادم من ألعاب الجوع	Literal translation
**00:06:38.64**	**Michael Fassbender**	Proper name	(مايكل فاسبندر)	Simple retention/transliteration + guidance (paranthesis)	مايكل فاسبندر	Simple retention/transliteration

**00:06:38.76**	He's doing the prequel to **the X-Men** prequel	Proper name	إنه يمثل في الجزء السابق **لفيلم (إكس مين)**	Simple retention/transliteration+ guidance (paranthesis)+ guidance (extra information)	هو يعمل على السابقة **لفيلم الإكس-مين**	Simple retention/transliteration+ add some guidance
**00:06:40.32**	**Jeremy Renner**	Proper name	ماذا عن **(جيريمي رينر)**؟	Simple retention/transliteration + guidance (paranthesis)	**جيريمي رينر؟**	Simple retention/transliteration
**00:06:46.0**	He's **the Hurt Locker guy**. He's **an Avenger**	Proper name Proper name	بطل **فيلم (هيرت لوكر)** إنه **ممثل في (أفنجرز)**	Simple retention/transliteration+ guidance (paranthesis)+ guidance (extra information) Simple retention/transliteration guidance (paranthesis)+ guidance (extra information) Simple	هو الرجل الذي عمل على **فيلم "The Hurt Locker"** هو أحد أبطال **فيلم "The Avengers"**	Guidance (extra information) +no translation Guidance (extra information) +no translation
**00:07:15.48**	And when we come back, an exclusive interview	Proper name	بعد قليل، لقاء حصري مع **(روبرت داونى) الابن**	Simple retention/transliteration + guidance (paranthesis)+	وعندما نعود، لقاء حصري **مع روبرت داونى جونيور**	Simple retention/transliteration

	with **Robert Downey Jr.**			standard transation		
**00:07:18.96**	Who's busy following up his billion-dollar **Iron Man** franchise	Proper name	المنهمك بعد النجاح الكبير الذي حققته سلسلة **(آيرون مان)**	Simple retention/transliteration + guidance (paranthesis)	الذي يعمل بجد على متابعة سلسلة **أفلام الرجل الحديدي** التي حققت مليارات الدولارات بنجاح	guidance (extra information) + standard translation
**00:07:22.04**	with the equally successful **Avengers** series	Proper name	مع سلسلة **(أفنجرز)** التي حصدت نفس القدر من النجاح	Simple retention/transliteration + guidance (paranthesis)	ع سلسلة **أفلام الأفنجرز** التي حققت نجاحًا مماثلاً	Simple retention/transliteration+ guidance (extra information)
**00:07:37.32**	and he's making a fortune in that **Tin Man getup**	Proper name	ومع ذلك فهو يجني ثروة من **ذلك الزي المعدني**	Minimum change/ literal translation	وهو يحقق ثروة من خلال ارتداء **زي الرجل الحديدي**	Standard translation
**00:08:51.40**	to adapting **Raymond Carver** for the stage	Proper name	لإعداد **رواية** **لـ(رايموند كارفر)** للمسرح؟	Simple retention/transliteration + guidance (extra information)	إلى **تكييف أعمال** **ريموند كارفر** للمسرح؟	Simple retention/transliteration+ guidance (extra information)
**00:09:20.32**	to Birdman, like **Icarus**	Proper name	(بيردمان)، شأنه شأن **(إيكاروس)**	Simple retention/transliteration + guidance (paranthesis)	إلى بيردمان، مثل **إيكاروس**	Simple retention/transliteration

**00:09:22.96**	Who is this **Barthes** guy?	Proper name	مهلاً... من هو **(بارث)**؟	Simple retention/transliteration+ guidance (paranthesis)	من هذا الرجل **بارث**؟	Simple retention/transliteration
**00:09:26.00**	**Roland Barthes** was a French philosopher	Proper name	**(رولاند بارث)** كان فيلسوفاً فرنسياً	Simple retention/transliteration+ guidance (paranthesis)	**رولان بارت** كان فيلسوفًا فرنسيًا	Simple retention/transliteration
**00:09:37.72**	It was tweeted by **@prostatewhispers**	Proper name	على **حساب (بروستايت ويسبرز) على (تويتر)**	Simple retention/transliteration guidance (paranthesis)+ guidance (extra information)	تم **التغريد** بواسطة **@prostatewhispers**.	Neologism +no translation
**00:10:18.88**	now want a **Times** feature	Proper name	إجراء مقال مطول مع **جريدة** الـ**(تايمز)**	Simple retention/transliteration guidance (paranthesis)+ guidance (extra information)	يريدون الآن مقالة في **تايمز**	Simple retention/transliteration
**00:12:43.24**	**Geraldine Page** **Helen Hayes,** **Jason Robards**	Proper name Proper name Proper name	**(جيرالدين بايج)** **(هيلين هايز)** **(جيسون روباردز)**	Simple retention/transliteration+ guidance (paranthesis) Simple retention/translite	جيرالدين بيج، هيلين هايز، جيسون روباردز	Simple retention/transliteration Simple retention/transliteration+

				ration+ guidance (paranthesis)<br><br>Simple retention/transliteration guidance (paranthesis)		Simple retention/transliteration
**00:12:47.00**	**Marlson Brando, and now, Riggan Thomson**	Proper name<br>Proper name	**(مارلون براندو)،** والآن **(ريغان توموسون)**	Simple retention/transliteration+ guidance (paranthesis)<br><br>Simple retention/transliteration+ guidance (paranthesis)	**مارلون براندو** والآن **ريجان طومسون**	Simple retention/transliteration+ Simple retention/transliteration
**00:16:40.04**	I saw you in **Hothouse** at **the Geffen**	Proper name	شاهدتك في **مسرحية (هاتهاوس)** في **مسرح (غيفين)**	Simple retention/transliteration + guidance (extra information)+ guidance (paranthesis)<br><br>Simple retention/transliteration + guidance (extra	**جيفن** رأيتك في **"Hothouse"**	Simple retention/transliteration +<br>No translation

				information)+ guidance (paranthesis)		
**00:18:29.32**	Listen, Mike... This is **Broadway**	Proper name	اسمع (مايك) هذه **(برودواى)**	Simple retention/transliteration + guidance (paranthesis)	استمع، مايك... هذه هي **برودواي**	Simple retention/transliteration
**00:23:39.12**	why aren't you drunk? This is **Carver**!	Proper name	هذه **مسرحية** **لـ(كارفر)** !	Retention+ guidance (extra information)+ guidance (paranthesis)	هذا هو **كارفر**	Simple retention/transliteration
**00:28:32.08**	**George Clooney** was sitting	Proper name	كان **(جورج كلونى)** يجلس	Simple retention/transliteration + guidance (paranthesis)	**جورج كلوني** كان جالساً	Simple retention/transliteration
**00:29:05.40**	it's going to be **Clooney**'s face on the front page, not mine	Proper name	سترى وجه **(كلونى)** في الصفحة الأولى، وليس وجهي	Simple retention/transliteration + guidance (paranthesis)	سيكون وجه **كلوني** على الصفحة الأمامية، ليس وجهي	Simple retention/transliteration
**00:29:17.00**	**Farrah Fawcett** died	Proper name	هل كنت تعرفين أن **(فرح فاوسيت)** ماتت	Simple retention/transliteration + guidance (paranthesis)	**فارا فوسيت** توفيت	Simple retention/transliteration

**00:29:18.92**	the exact same day as **Michael Jackson**	Proper name	في نفس اليوم الذي مات فيه **(مايكل جاكسون)**	Simple retention/transliteration + guidance (paranthesis)	في نفس اليوم تمامًا كما **مايكل جاكسون**.	Simple retention/transliteration
**00:29:49.12**	you did with **Goldie Hawn**	Proper name	الذي مثلته مع **(غولدي هون)**	Simple retention/transliteration + guidance (paranthesis)	التي قمت بها مع **جولدي هون**	Simple retention/transliteration
**00:30:04.92**	You're not **Farrah Fawcett**	Proper name	أنت لست **(فرح فاوسيت)**	Simple retention/transliteration + guidance (paranthesis)	أنت لست **فارا فوسيت**	Simple retention/transliteration
**00:30:09.36**	We should have done that reality show they offered us **The Thomsons**	Proper name	كان علينا إنتاج ذلك المسلسل الواقعي، **(آل تومسون)**	Simple retention/transliteration + guidance (paranthesis)	كان يجب أن نفعل ذلك البرنامج الواقعي الذي عرضوه علينا **"The Thomsons".**	No translation
**00:31:04.00**	who goes and tells coy, slightly vomitous stories on **Letterman**	Proper name	ويروي قصصا خجولة ومقززة بعض الشيء في **استعراض (ليترمان)**	Simple retention/transliteration + guidance (paranthesis)+ guidance (extra information)	يذهب ويروي قصصاً مترفة، قليلاً مقرفة، على **برنامج ليترمان**	Simple retention/transliteration + guidance (extra information)

**00:31:33.28**	That was **P.T. Barnum**'s premise when he invented the circus	Proper name	هذه كانت فكرة **(بي تي بارنوم)** عندما ابتكر السيرك	Simple retention/transliteration + guidance (paranthesis)	هذا كان **فكر بي. تي. بارنوم** عندما اخترع السيرك	Simple retention/transliteration
**00:31:57.08**	this is **New York** City	Proper name	أنت في مدينة **(نيويورك)**	Simple retention/transliteration + guidance (paranthesis)	هذه مدينة **نيويورك**	Simple retention/transliteration
**00:32:48.60**	in 500 words in **the Times**	Proper name	بخمسمائة كلمة في **الـ(تايمز)**	Simple retention/transliteration+ guidance (paranthesis)	في 500 كلمة في **صحيفة تايمز**	Simple retention/transliteration + guidance (extra information)
**00:32:51.56**	the only opinion that matters in **New York** theater is hers	Proper name	رأيها هو الوحيد المهم في الوسط المسرحي في **(نيويورك)**	Simple retention/transliteration+ guidance (paranthesis)	الرأي الوحيد الذي يهم في مسرح **نيويورك** هو رأيها	Simple retention/transliteration
**00:34:06.16**	doing a play in **Syracuse**	Proper Name	أمثل في مسرحية في **(سيراكيوز)**	Simple retention/transliteration+ guidance (paranthesis)	أؤدي مسرحية في **سيراكيوز**	Simple retention/transliteration
**00:34:58.20**	That's **Flaubert**, right?	Proper name	إنه **اقتباس** من **(فلوبير)**، صحيح؟	Simple retention/transliteration guidance (paranthesis)+	هذا **فلوبير**، أليس كذلك؟	Simple retention/transliteration

				guidance (extra information)		
**00:44:32.24**	I’ve always dreamed of being a Broadway actress	Proper name	لطالما حلمت بأن أصبح ممثلة في **(برودواي)**	Simple retention/transliteration+ guidance (paranthesis)	لقد حلمت دائمًا بأن أكون ممثلة على **برودواي**.	Simple retention/transliteration
**00:48:04.16**	that dude from **American Pie** was there	Proper name	ممثل **(أميريكان باى)** كان هناك	Simple retention/transliteration+ guidance (paranthesis)	تلك الشخصية من **فيلم الفطيرة الأمريكي** كان هناك.	Literal translation+ guidance (extra information)
**00:55:57.56**	You think you can get fucking **Ryan Gosling**	Proper name	هل ستستعين **بـ(راين غوزلينغ)**	Simple retention/transliteration+ guidance (paranthesis)	هل تعتقد أنك يمكنك الحصول على **رايان غوسلين** لعنته؟	Simple retention/transliteration
**00:56:01.36**	What do you think my friend, Tabitha is going to do to you in **the Times**?	Proper name	ماذا تظن أن صديقتي (تابيثا) ستفعل بك على صفحات الـ**(تايمز)**	Simple retention/transliteration+ guidance (paranthesis)+ guidance (extra information)	ما الذي تعتقد يا صديقي، تابيثا ستفعله لك في **الصحيفة**؟	Omission (but transfer the sense by other means)
**01:00:21.04**	**Martin Scorsese**	Proper name	**(مارتن سكورسيز)**	Simple retention/transliteration + guidance (paranthesis)	**مارتن سكورسيزي**	Simple retention/transliteration

**01:04:49.72**	**Oprah**<br>**Hallmark**<br>**R. Kelly bad**	Proper name<br>Proper name<br>Proper name	**(أوبرا)**<br>**بطاقات (هولمارك)**<br>و سيء كـ**(آر كيلي)**	Simple retention/transliteration + guidance (paranthesis)<br>Simple retention/transliteration+ guidance (paranthesis)+ guidance (extra information)<br>Simple retention/transliteration+ guidance (paranthesis)	**أوبرا**<br>**هولمارك**<br>**آر كيلي**سيء	Simple retention/transliteration<br>Simple retention/transliteration<br>Simple retention/transliteration+ literal translation
**01:08:31.04**	Or **Justin Bieber**	Proper name	أو **شبيه (جاستن بيبر)**	Simple retention/transliteration + guidance (paranthesis)+ guidance (extra information)	أو **جاستن بيبر**	Simple retention/transliteration
**01:27:02.88**	Flames, sacrifice... **lcarus**	Proper name	نيران تضحية<br>**(إيكاروس)**	Simple retention/transliteration +paranthesis	اللهب، التضحية...<br>**إيكاروس**	Simple retention/transliteration
**01:41:08.52**	We'll use **Meg Ryan's guy**	Proper name	سنستعين **بطبيب التجميل الخاص** بـ**(ميغ راين)**	Simple retention/transliteration + guidance (paranthesis)+	سنستخدم الرجل **المقرب من ميغ رايان**	Simple retention/transliteration +

				guidance (extra information)		guidance (extra information)
